# Mortality of Philadelphia for April, May and June, 1856; Collated from the Health Office Record

**Published:** 1856-08

**Authors:** Wilson Jewell


*Mortality of Philadelphia for April, May and June*, 1856; *collated from the Health Office record.* By WILSON JEWELL, M. D.

The mortality of our city for the second quarter of the year, ending June 28th, amounted to 2567. This includes a period of ninety days, and averages 28½ deaths per day. According to this statement, there has been an increase of 2½ deaths per day, over those for the like quarter of 1855. Nevertheless, the city has been healthy. The only prevailing diseases have been those of the exanthematous fevers, Scarlatina, Measles and Small Pox, the latter of which has been rapidly declining.

Measles have been very prevalent, but uncomplicated and mild in their character.

The number of deaths from diseases alone, have been 2099; the remainder were the result of Still Born, Old Age, External Causes, Debility, Malformation and Unknown.

Among males, the deaths numbered 1320; of females, 1251; exhibiting an excess of 2·67 per cent. in the male sex.

The mortality among children under one year of age (exclusive of Still Born,) was 532, equal to 20·72 per cent. Under five years 1057, or 42·34 per cent.

The excess of male Still Born children was equal to 6 per cent. for the quarter.

Of the "Endemics," the mortality was 542. A decided increase over those for the like quarter of 1855, equal to 19·25 per cent. This increase is easily accounted for, by the addition to the deaths this year from Scarlet Fever and Small Pox; the former numbering 186; the latter 99.

By comparing the deaths from Small Pox with those of the first quarter of the present year, a large falling off will be observed, amounting to 41 per cent. An evidence of the decline of the disease.

Of the deaths from this class of diseases, 418, or 77 per cent. were among children under ten years of age. Of the deaths from Small Pox, 62 were under ten years, and of those from Scarlet Fever, 178 were under ten years.

Under the classification of "Uncertain or General Seat," the deaths from which amount to 338, we would notice particularly, those recorded under the cognomen Debility, numbering 100, and Marasmus 78; constituting more than half of all the deaths under this head. We cannot believe that Debility is a disease, any more than is its correlative, strength or vigor. It is only a symptom, or a certain result, in infants especially, of an imperfect developement of the organic or functional system; or else is produced by morbid agents, acting either upon the skin, alimentary canal or respiratory tissues, resulting in diseased action, and involving other and more vital functions. A careful diagnosis would, in almost every instance, lead to a more correct result, and enable the practitioner to avoid the too frequent and too familiar use, with many at least, of the vague and unmeaning term debility, as a cause of death. Almost as much might be urged against the application of the term Marasmus, which will be found in frequent use in mortuary statistics, and in this instance accounts for 78 deaths; fifty-three of which were in children under one year of age. It is true, however, that the meaning of this word is more generally understood and more definitely applied, but we are of opinion, that by far in too many instances, as in the use of debility, it is employed at random, or as a kind of scape-goat for ignorance of the true nature of the disease, or indolence to investigate its seat. In short, if the profession at large would discriminate more closely when certifying to the cause of death, we should possess a far more perfect statistical analysis of diseases, and find but comparatively little need of the classification "Uncertain."

The deaths from diseases of the "Nervous System," partake of but little variation, numerically, from one quarter to the other. This second quarter sums up 444; of this number 309, or 69 per cent. were under five years of age. Convulsions (what cause?) furnishes 140 of the deaths, and Inflammation of the Brain 72. Under this classification, the excess of deaths among males is equal to 9·45 per cent.

The "Organs of Respiration" are charged with 623 deaths; nearly one-fourth of the whole number for the quarter. As usual, Consumption of the Lungs furnishes a large quota, nearly 61 per cent. Compared with those for last year, second quarter, there will be found an increase of 7 per cent. While a comparison of all the deaths under this head with those of the same period last year, will show a decrease of only one death in the aggregate.

Thirty-four deaths have occurred during the quarter, from Diseases of the Heart. Forty-three from Inflammation of the Stomach and Bowels. Twenty from Puerperal Fever. One hundred and nineteen from External Causes. Thirty-six from Old Age. Forty-one from Unknown Causes, and one hundred and sixty-five were Still Born.

Of children there died 1507; adults 1064.

The highest number of deaths in any one week was 219. The lowest, 159. The average of deaths weekly was 197¾.

TABLE NO. I.

*Deaths for the second quarter of 1856 classified.*

| | Ap. | Ma. | Jun. | Male. A. | Male. M. | Male. J. | Female. A. | Female. M. | Female. J. | Total. |
|---|---|---|---|---|---|---|---|---|---|---|
| 1 *Endemic & Contagious diseases* | | | | | | | | | | |
| Zymotic or Epidemic | 205 | 138 | 199 | 96 | 72 | 102 | 109 | 66 | 97 | 542 |
| 2 *Uncertain or general seat,* | | | | | | | | | | |
| Sporadic diseases | 128 | 103 | 107 | 73 | 49 | 55 | 55 | 54 | 52 | 338 |
| 3 Nervous system | 177 | 124 | 143 | 101 | 61 | 81 | 76 | 63 | 62 | 444 |
| 4 Organs of Respiration | 299 | 173 | 151 | 159 | 80 | 73 | 140 | 93 | 78 | 623 |
| 5 " Circulation | 27 | 18 | 19 | 16 | 8 | 9 | 11 | 10 | 10 | 64 |
| 6 Digestive organs | 50 | 38 | 35 | 15 | 18 | 23 | 35 | 20 | 12 | 123 |
| 7 Urinary " | 7 | 5 | 5 | 3 | 3 | 3 | 4 | 2 | 2 | 17 |
| 8 Organs of Generation | 11 | 13 | 10 | | | | 11 | 13 | 10 | 34 |
| 9 " Locomotion | 8 | 2 | 7 | 6 | | 6 | 2 | 2 | 1 | 17 |
| 10 Integumentary system | 1 | 1 | 4 | 1 | 1 | 3 | | | 1 | 6 |
| 11 Old age | 15 | 11 | 10 | 4 | 3 | 3 | 11 | 8 | 7 | 36 |
| 12 External causes | 46 | 42 | 33 | 30 | 25 | 22 | 16 | 17 | 11 | 121 |
| Still Born | 60 | 58 | 47 | 31 | 30 | 27 | 29 | 28 | 20 | 165 |
| Unknown | 18 | 10 | 13 | 13 | 7 | 8 | 5 | 3 | 5 | 41 |
| | 1052 | 736 | 783 | 548 | 357 | 415 | 504 | 379 | 368 | 2571 |

### TABLE NO. 2.

1. *Endemic and Contagious Diseases—Zymotic or Epidemic.*

| | Male. | Female. | Under 1 yr. | 1 to 2. | 2 to 5. | 5 to 10. | 10 to 15. | 15 to 20. | 20 to 30. | 30 to 40. | 40 to 50. | 50 to 60. | 60 to 70. | 70 to 80. | 80 to 90. | 90 to 100. | 100 to 110. | Total. |
|---|---|---|---|---|---|---|---|---|---|---|---|---|---|---|---|---|---|---|
| Cholera | 1 | 1 | | | | 2 | | | | | | | | | | | | 2 |
| " infantum | 16 | 16 | 29 | 3 | | | | | | | | | | | | | | 32 |
| " morbus | 2 | 1 | 1 | | | 1 | | | | | 1 | | | | | | | 3 |
| Croup | 21 | 23 | 6 | 13 | 21 | 4 | | | | | | | | | | | | 44 |
| Diarrhœa | 15 | 8 | 11 | 2 | 3 | | | 1 | 1 | | 2 | 1 | 2 | | | | | 23 |
| Dysentery | 12 | 8 | 6 | 6 | 2 | | | | | 1 | 1 | 1 | | 2 | 1 | | | 20 |
| Erysipelas | 13 | 12 | 6 | | 1 | | | 1 | 4 | 2 | 5 | 3 | 1 | 1 | 1 | | | 25 |
| Fever, | 1 | 1 | | | 1 | | | | | | | | 1 | | | | | 2 |
| " Bilious | 5 | | 1 | | | | | | 1 | | | 1 | | 2 | | | | 5 |
| " Congestive | 1 | 1 | | | | | | 1 | 1 | | | | | | | | | 2 |
| " Gastric | 1 | 1 | | 1 | 1 | | | | | | | | | | | | | 2 |
| " Inflammatory | 1 | | | | | | 1 | | | | | | | | | | | 1 |
| " Intermittent | 3 | | | 1 | | 1 | | 1 | | | | | | | | | | 3 |
| " Nervous | 1 | | | | | 1 | | | | | | | | | | | | 1 |
| " Remittent | 4 | 3 | | 3 | 2 | 1 | | | | | | 1 | | | | | | 7 |
| " Scarlet | 89 | 97 | 19 | 37 | 91 | 31 | 4 | 3 | | 1 | | | | | | | | 186 |
| " Typhoid | 15 | 24 | | 1 | 2 | 5 | | 5 | 9 | 8 | 3 | 3 | 3 | | | | | 39 |
| " Typhus | | 2 | | | | | | 1 | | 1 | | | | | | | | 2 |
| Hooping Cough | 10 | 11 | 9 | 8 | 4 | | | | | | | | | | | | | 21 |
| Influenza | | 1 | | | | | | | | | | | | 1 | | | | 1 |
| Measles | 4 | 9 | 1 | 3 | 7 | 2 | | | | | | | | | | | | 13 |
| Small Pox | 50 | 49 | 17 | 11 | 22 | 12 | 3 | 4 | 8 | 15 | 6 | | | 1 | | | | 99 |
| Syphilis | 2 | | 1 | | | | | | 1 | | | | | | | | | 2 |
| Varioloid | 3 | 4 | 3 | 1 | 1 | | | | | 1 | 1 | | | | | | | 7 |
| | 270 | 272 | 110 | 90 | 158 | 60 | 8 | 17 | 25 | 29 | 19 | 10 | 7 | 7 | 2 | | | 542 |

### 2. *Uncertain or General Seat—Sporadic Diseases.*

| | Male. | Female. | Under 1 yr. | 1 to 2. | 2 to 5. | 5 to 10. | 10 to 15. | 15 to 20. | 20 to 30. | 30 to 40. | 40 to 50. | 50 to 60. | 60 to 70. | 70 to 80. | 80 to 90. | 90 to 100. | 100 to 110. | Total. |
|---|---|---|---|---|---|---|---|---|---|---|---|---|---|---|---|---|---|---|
| Abscess | 3 | 3 | 1 | | | | 1 | | | 2 | 1 | | | 1 | | | | 6 |
| " Lumbar | | 1 | | | | | | | 1 | | | | | | | | | 1 |
| " Psoas | 1 | 1 | | | | | | | 2 | | | | | | | | | 2 |
| Anasarca | | 1 | | | 1 | | | | | | | | | | | | | 1 |
| Angina | | 1 | | 1 | | | | | | | | | | | | | | 1 |
| Cancer | 8 | 9 | 1 | | | | | | | 5 | 1 | 4 | 4 | 2 | | | | 17 |
| Carcinoma | 1 | 1 | | | | 1 | | | | | | 1 | | | | | | 2 |
| " of Breast | | 1 | | | | | | | | 1 | | | | | | | | 1 |
| Congestion | | 1 | | | | | | | | | | | | | 1 | | | 1 |
| Cyanosis | 7 | 6 | 12 | | | | | | | | | 1 | | | | | | 13 |
| Debility | 55 | 45 | 46 | 2 | 3 | | | | | 6 | 6 | 6 | 12 | 12 | 7 | | | 100 |
| Disease of Throat | | 1 | | | | | | 1 | | | | | | | | | | 1 |
| Dropsy | 21 | 16 | 1 | 1 | 5 | 2 | 2 | | 4 | 2 | 5 | 4 | 4 | 6 | 1 | | | 37 |
| Fungus Hæmatodes | 2 | | | | | | | | | 2 | | | | | | | | 2 |
| Gangrene | 2 | 2 | | | 2 | | | | 1 | | | | | 1 | | | | 4 |
| Gout | 1 | | | | | | | | | | | 1 | | | | | | 1 |
| Hemorrhage | 5 | 10 | 2 | | 1 | | 1 | | 6 | 2 | 1 | 1 | | 1 | | | | 15 |
| Inanition | 7 | 1 | 7 | | | | | | | | | 1 | | | | | | 8 |
| Inflammation | 2 | 1 | | 1 | | | | | 1 | | 1 | | | | | | | 3 |
| " Breast | 1 | | 1 | | | | | | | | | | | | | | | 1 |
| " Leg | 2 | | | | | | | | | | | | 1 | | 1 | | | 2 |
| " Throat | 1 | 4 | 2 | | 2 | | | | | 1 | | | | | | | | 5 |
| " Tonsils | | 1 | | | | | | | | 1 | | | | | | | | 1 |
| Malformation | 4 | 3 | 7 | | | | | | | | | | | | | | | 7 |
| Marasmus | 38 | 40 | 53 | 8 | 5 | 2 | 2 | | 2 | 1 | 1 | 1 | 1 | 2 | | | | 78 |
| Ramollissement | | 1 | | | | | | | | 1 | | | | | | | | 1 |
| Scirrhus | 1 | | | | | | | | | | 1 | | | | | | | 1 |
| Scrofula | 10 | 7 | 2 | 5 | 4 | 2 | | 2 | 1 | | | 1 | | | | | | 17 |
| Tabes Mesenterica | 5 | 3 | 4 | 1 | | 1 | | 1 | 1 | | | | | | | | | 8 |
| Ulceration of Throat | | 1 | | | | | | | | | | | | 1 | | | | 1 |
| | 177 | 161 | 139 | 19 | 23 | 8 | 6 | 4 | 19 | 24 | 17 | 21 | 22 | 26 | 10 | | | 338 |

### 3. *Nervous Diseases.*

| | Male. | Female. | Under 1 yr. | 1 to 2. | 2 to 5. | 5 to 10. | 10 to 15. | 15 to 20. | 20 to 30. | 30 to 40. | 40 to 50. | 50 to 60. | 60 to 70. | 70 to 80. | 80 to 90. | 90 to 100. | 100 to 110. | Total. |
|---|---|---|---|---|---|---|---|---|---|---|---|---|---|---|---|---|---|---|
| Apoplexy | 18 | 14 | | | | 1 | | | 1 | 1 | 5 | 4 | 9 | 6 | 4 | 1 | | 32 |
| Catalepsy | 1 | | | 1 | | | | | | | | | | | | | | 1 |
| Chorea | 1 | 1 | | | | 1 | | | | | | | 1 | | | | | 2 |
| Compression of Brain | | 1 | | 1 | | | | | | | | | | | | | | 1 |
| Congestion " | 20 | 19 | 10 | 3 | 6 | 3 | 1 | 1 | 3 | 4 | 5 | | | 1 | 2 | | | 39 |
| Convulsions | 78 | 62 | 83 | 23 | 23 | 3 | 3 | | 3 | 1 | 1 | | | | | | | 140 |
| Cramp | 2 | | 1 | | 1 | | | | | | | | | | | | | 2 |
| Disease of Brain | 14 | 9 | 7 | 4 | 3 | 1 | 1 | 1 | | 2 | 3 | | | 1 | | | | 23 |
| Dropsy " | 25 | 27 | 22 | 10 | 13 | 4 | 1 | | | | | | 2 | | | | | 52 |
| Effusion " | 15 | 12 | 10 | 7 | 1 | 2 | | 1 | 1 | 2 | 1 | 1 | 1 | | | | | 27 |
| Epilepsy | | 2 | 1 | | | | 1 | | | | | | | | | | | 2 |
| Inflammation of Brain | 39 | 33 | 21 | 13 | 15 | 9 | 3 | | 7 | 2 | 1 | 1 | | | | | | 72 |
| Mania | 1 | 1 | | | | | | | | 1 | | | 1 | | | | | 2 |
| " a Potu | 6 | 1 | | | | | | | 1 | 3 | 2 | 1 | | | | | | 7 |
| Palsy | 12 | 16 | | | 1 | | | | | 2 | 1 | 6 | 9 | 6 | 3 | | | 28 |
| Softening of Brain | 6 | 3 | 2 | | 1 | 2 | | | 1 | | 1 | | | 1 | 1 | | | 9 |
| Sun Stroke | 1 | | | | | | | | 1 | | | | | | | | | 1 |
| Tetanus | 4 | | | | | | | 1 | | | 2 | 1 | | | | | | 4 |
| | 243 | 201 | 157 | 62 | 64 | 26 | 10 | 4 | 18 | 18 | 22 | 14 | 23 | 15 | 10 | 1 | | 444 |

### 4. *Organs of Respiration.*

| | Male. | Female. | Under 1 yr. | 1 to 2. | 2 to 5. | 5 to 10. | 10 to 15. | 15 to 20. | 20 to 30. | 30 to 40. | 40 to 50. | 50 to 60. | 60 to 70. | 70 to 80. | 80 to 90. | 90 to 100. | 100 to 110. | Total. |
|---|---|---|---|---|---|---|---|---|---|---|---|---|---|---|---|---|---|---|
| Asthma | 2 | 3 | 1 | | | | | | | 1 | 1 | | | 1 | 1 | | | 5 |
| Collapse of Lungs | | 1 | 1 | | | | | | | | | | | | | | | 1 |
| Congestion " | 24 | 8 | 8 | 3 | 5 | 3 | 1 | 1 | 1 | 4 | 1 | 2 | 3 | | | | | 32 |
| Consumption " | 176 | 204 | 10 | 11 | 25 | 3 | 6 | 23 | 126 | 81 | 44 | 30 | 14 | 6 | 1 | | | 380 |
| Disease of " | 7 | 5 | 1 | 1 | 3 | | | 1 | 1 | 1 | 1 | | 2 | 1 | | | | 12 |
| " Chest | | 2 | | | | | | | 1 | | | 1 | | | | | | 2 |
| Dropsy of " | 8 | 7 | 1 | | 1 | 2 | | | | 1 | 2 | 2 | 1 | 4 | 1 | | | 15 |
| Effusion of " | 4 | 1 | | | 1 | | 1 | | | | | | 2 | 1 | | | | 5 |
| Empyema | 1 | | | | | | | | | | | | 1 | | | | | 1 |
| Hemorrhage of Lungs | 6 | | 1 | | | | | | 2 | 1 | 2 | | | | | | | 6 |
| Inflamm'n of Bronchi | 29 | 28 | 24 | 8 | 9 | 1 | 1 | | 3 | | | 2 | 2 | 5 | 2 | | | 57 |
| " Chest | | 1 | | 1 | | | | | | | | | | | | | | 1 |
| " Larynx | 4 | 4 | 1 | 1 | 2 | 2 | | | 1 | 1 | | | | | | | | 8 |
| " Lungs | 48 | 44 | 25 | 11 | 11 | 5 | | 3 | 7 | 8 | 6 | 5 | 6 | 5 | | | | 92 |
| " Pleura | 2 | 3 | | | 1 | | | | | 1 | 1 | 1 | 1 | | | | | 5 |
| Ulceration of Larynx | 1 | | | 1 | | | | | | | | | | | | | | 1 |
| | 312 | 311 | 73 | 37 | 58 | 16 | 9 | 28 | 142 | 99 | 58 | 43 | 32 | 23 | 5 | | | 623 |

### 5. *Organs of Circulation.*

| | Male. | Female. | Under 1 yr. | 1 to 2. | 2 to 5. | 5 to 10. | 10 to 15. | 15 to 20. | 20 to 30. | 30 to 40. | 40 to 50. | 50 to 60. | 60 to 70. | 70 to 80. | 80 to 90. | 90 to 100. | 100 to 110. | Total. |
|---|---|---|---|---|---|---|---|---|---|---|---|---|---|---|---|---|---|---|
| Angina Pectoris | 2 | 1 | | | 1 | | | | | | 1 | | 1 | | | | | 3 |
| Anæmia | | 3 | | 1 | | | | | 1 | 1 | | | | | | | | 3 |
| Aneurism of Aorta | 1 | | | | | | | | 1 | | | | | | | | | 1 |
| Disease of Heart | 22 | 12 | 2 | 1 | 1 | | 2 | 2 | 6 | | 5 | 6 | 5 | 4 | | | | 34 |
| Dropsy " | 1 | 3 | | | | | | | 1 | 1 | 1 | | 1 | | | | | 4 |
| Enlargement of Heart | 2 | 7 | | 1 | | 2 | | 2 | 1 | 1 | 1 | | | 1 | | | | 9 |
| Fatty degeneration " | | 1 | | | | | | | | | | | | | | 1 | | 1 |
| Gout of " | 1 | | | | | | | | | | | | | 1 | | | | 1 |
| Inflammation of " | 2 | 3 | | | 1 | 1 | 1 | | | 1 | 1 | | | | | | | 5 |
| Rupture of Blood-vessels | 1 | 1 | | | | | | | | 1 | 1 | | | | | | | 2 |
| " Veins | 1 | | | | | | | | | | | | 1 | | | | | 1 |
| | 33 | 31 | 2 | 3 | 3 | 3 | 3 | 4 | 10 | 5 | 10 | 6 | 8 | 6 | | 1 | | 64 |

### 6. *Digestive Organs.*

| | Male. | Female. | Under 1 yr. | 1 to 2. | 2 to 5. | 5 to 10. | 10 to 15. | 15 to 20. | 20 to 30. | 30 to 40. | 40 to 50. | 50 to 60. | 60 to 70. | 70 to 80. | 80 to 90. | 90 to 100. | 100 to 110. | Total. |
|---|---|---|---|---|---|---|---|---|---|---|---|---|---|---|---|---|---|---|
| Cancrum Oris | 1 | 1 | | | 1 | 1 | | | | | | | | | | | | 2 |
| Carcinoma of Liver | | 1 | | | | | | | 1 | | | | | | | | | 1 |
| " Stomach | 1 | | | | | | | | | | | 1 | | | | | | 1 |
| Cirrhosis of Liver | 2 | 1 | | | | | | | 1 | | 1 | 1 | | | | | | 3 |
| Congestion of " | 1 | 5 | 2 | | 1 | | | | | | 1 | | 2 | | | | | 6 |
| " Stomach | | 1 | | 1 | | | | | | | | | | | | | | 1 |
| Disease of Liver | 4 | 4 | | | | 1 | | | 2 | 2 | | 2 | 1 | | | | | 8 |
| " Stom. & Bowels | 2 | 2 | 2 | | | | | | 1 | | | 1 | | | | | | 4 |
| Dropsy of Abdomen | | 1 | | | | | | | | | | | | 1 | | | | 1 |
| Inflammat. Abdomen | | 1 | | | | | | | | | | 1 | | | | | | 1 |
| " Liver | 6 | 4 | | 1 | | | 1 | | | 1 | 2 | 2 | 2 | 1 | | | | 10 |
| " Peritoneum | 5 | 10 | | | | 1 | | | 6 | 3 | | 3 | 1 | 1 | | | | 15 |
| " Stom. & Bowels | 20 | 23 | 11 | 4 | 5 | 4 | 2 | 1 | 4 | 5 | 1 | 1 | 3 | 2 | | | | 43 |
| Intussusception | 1 | | 1 | | | | | | | | | | | | | | | 1 |
| Jaundice | 5 | 5 | 4 | | | | | | 1 | 1 | | 2 | 2 | | | | | 10 |
| Obstruction of Bowels | 1 | | | | | | | 1 | | | | | | | | | | 1 |
| Perforation of Bowels | 1 | | | | | | | | | 1 | | | | | | | | 1 |
| Scirrhus of Stomach | | 2 | | | | | | | 1 | | | | | 1 | | | | 2 |
| Softening " | 1 | | 1 | | | | | | | | | | | | | | | 1 |
| Teething | 2 | 4 | 3 | 3 | | | | | | | | | | | | | | 6 |
| Tympanitis | | 1 | | | | | | | | 1 | | | | | | | | 1 |
| Ulceration of Bowels | 1 | | | | 1 | | | | | | | | | | | | | 1 |
| " Stomach | 1 | | | | | | | | | 1 | | | | | | | | 1 |
| " Rectum | | 1 | | | | | | | 1 | | | | | | | | | 1 |
| Worms | 1 | | | | 1 | | | | | | | | | | | | | 1 |
| | 56 | 67 | 24 | 9 | 9 | 7 | 3 | 2 | 18 | 15 | 5 | 14 | 11 | 6 | | | | 123 |

### 7. *Urinary Organs.*

| | Male. | Female. | Under 1 yr. | 1 to 2. | 2 to 5. | 5 to 10. | 10 to 15. | 15 to 20. | 20 to 30. | 30 to 40. | 40 to 50. | 50 to 60. | 60 to 70. | 70 to 80. | 80 to 90. | 90 to 100. | 100 to 110. | Total. |
|---|---|---|---|---|---|---|---|---|---|---|---|---|---|---|---|---|---|---|
| Albuminuria | 3 | 3 | | | | 1 | | 1 | 1 | 1 | 1 | | | 1 | | | | 6 |
| Diabetes | 1 | | | | | | | | 1 | | | | | | | | | 1 |
| Disease of Kidneys | 3 | 1 | | | | | 1 | | | 1 | | | | 2 | | | | 4 |
| Inflammation of Bladder | 1 | 3 | | | | 1 | | | 1 | 1 | | | 1 | | | | | 4 |
| " Kidneys | 1 | 1 | | | | | | | | | | | 1 | | | 1 | | 2 |
| | 9 | 8 | | | | 2 | 1 | 1 | 3 | 3 | 1 | | 2 | 3 | | 1 | | 17 |

### 8. *Organs of Generation.*

| | Male. | Female. | Under 1 yr. | 1 to 2. | 2 to 5. | 5 to 10. | 10 to 15. | 15 to 20. | 20 to 30. | 30 to 40. | 40 to 50. | 50 to 60. | 60 to 70. | 70 to 80. | 80 to 90. | 90 to 100. | 100 to 110. | Total. |
|---|---|---|---|---|---|---|---|---|---|---|---|---|---|---|---|---|---|---|
| Amenorrhœa | | 1 | | | | | | | | 1 | | | | | | | | 1 |
| Carcinoma of Uterus | | 1 | | | | | | | | 1 | | | | | | | | 1 |
| Disease of " | | 1 | | | | | | | 1 | | | | | | | | | 1 |
| Hemorrhage " | | 1 | | | | | | | | 1 | | | | | | | | 1 |
| Inflammation of " | | 6 | | | | | | | 2 | 3 | 1 | | | | | | | 6 |
| Ovarian Dropsy | | 1 | | | | | | | | 1 | | | | | | | | 1 |
| " Tumour | | 1 | | | | | | | | | 1 | | | | | | | 1 |
| Puerperal Fever, | | 20 | | | | | | | 11 | 9 | | | | | | | | 20 |
| Tumour of Uterus | | 2 | | | | 1 | | | | | 1 | | | | | | | 2 |
| | | 34 | | | | 1 | | | 14 | 16 | 3 | | | | | | | 34 |

### 9. *Organs of Locomotion.*

| | Male. | Female. | Under 1 yr. | 1 to 2. | 2 to 5. | 5 to 10. | 10 to 15. | 15 to 20. | 20 to 30. | 30 to 40. | 40 to 50. | 50 to 60. | 60 to 70. | 70 to 80. | 80 to 90. | 90 to 100. | 100 to 110. | Total. |
|---|---|---|---|---|---|---|---|---|---|---|---|---|---|---|---|---|---|---|
| Caries | 2 | 1 | | | | | 1 | | 1 | | 1 | | | | | | | 3 |
| " of Spine | | 1 | | | | | | | 1 | | | | | | | | | 1 |
| Disease of Hip | 1 | | | | | 1 | | | | | | | | | | | | 1 |
| " Spine | 2 | | | | | 2 | | | | | | | | | | | | 2 |
| Inflammation " | | 1 | | | 1 | | | | | | | | | | | | | 1 |
| Rheumatism | 7 | 1 | | | | 3 | | 1 | | 1 | | | | 3 | | | | 8 |
| Softening of Spinal Marrow | 1 | | | | | 1 | | | | | | | | | | | | 1 |
| | 13 | 4 | | | 1 | 7 | 1 | 1 | 2 | 1 | 1 | | | 3 | | | | 17 |

### 10. *Integumentary System.*

| | Male. | Female. | Under 1 yr. | 1 to 2. | 2 to 5. | 5 to 10. | 10 to 15. | 15 to 20. | 20 to 30. | 30 to 40. | 40 to 50. | 50 to 60. | 60 to 70. | 70 to 80. | 80 to 90. | 90 to 100. | 100 to 110. | Total. |
|---|---|---|---|---|---|---|---|---|---|---|---|---|---|---|---|---|---|---|
| Anthrax | 1 | | | | | | | | | | | | 1 | | | | | 1 |
| Purpura | 1 | | | | | | 1 | | | | | | | | | | | 1 |
| " Hemorrhagica | 3 | 1 | 1 | 1 | 1 | 1 | | | | | | | | | | | | 4 |
| | 5 | 1 | 1 | 1 | 1 | 1 | 1 | | | | | | 1 | | | | | 6 |

### 11. *Old Age.*

| | Male. | Female. | Under 1 yr. | 1 to 2. | 2 to 5. | 5 to 10. | 10 to 15. | 15 to 20. | 20 to 30. | 30 to 40. | 40 to 50. | 50 to 60. | 60 to 70. | 70 to 80. | 80 to 90. | 90 to 100. | 100 to 110. | Total. |
|---|---|---|---|---|---|---|---|---|---|---|---|---|---|---|---|---|---|---|
| Old Age | 10 | 26 | | | | | | | | | | | 1 | 11 | 17 | 7 | | 36 |

### 12. *External Causes.*

| | Male. | Female. | Under 1 yr. | 1 to 2. | 2 to 5. | 5 to 10. | 10 to 15. | 15 to 20. | 20 to 30. | 30 to 40. | 40 to 50. | 50 to 60. | 60 to 70. | 70 to 80. | 80 to 90. | 90 to 100. | 100 to 110. | Total. |
|---|---|---|---|---|---|---|---|---|---|---|---|---|---|---|---|---|---|---|
| Asphyxia | 4 | 6 | 8 | | | | | | | | | 1 | 1 | | | | | 10 |
| Burns | 6 | 6 | | 2 | 3 | 1 | | 3 | 1 | 1 | | | 1 | | | | | 12 |
| Casualties | 26 | 9 | 4 | 2 | 3 | 2 | | 1 | 9 | 6 | 3 | 3 | 1 | 1 | | | | 35 |
| Drowned | 23 | 5 | | 1 | 1 | 1 | 7 | 2 | 7 | 3 | 5 | 1 | | | | | | 28 |
| Exhaustion | 2 | 5 | | | | | | | 2 | 1 | 3 | 1 | | | | | | 7 |
| Exposure | | 1 | 1 | | | | | | | | | | | | | | | 1 |
| Fracture | 2 | | | | | | | | | | 1 | | 1 | | | | | 2 |
| " Skull | 1 | 1 | | | | | | | 1 | | | | 1 | | | | | 2 |
| Intemperance | 5 | 6 | | | | | | | | 4 | 6 | | 1 | | | | | 11 |
| Poisoning | 1 | | 1 | | | | | | | | | | | | | | | 1 |
| Strangulation | | 1 | 1 | | | | | | | | | | | | | | | 1 |
| Suffocation | 1 | | 1 | | | | | | | | | | | | | | | 1 |
| Suicide | 6 | 4 | | | | | | 1 | 2 | 2 | 3 | 2 | | | | | | 10 |
| | 77 | 44 | 16 | 5 | 7 | 4 | 7 | 7 | 22 | 17 | 21 | 8 | 6 | 1 | | | | 121 |

| | Male. | Female. | Under 1 yr. | 1 to 2. | 2 to 5. | 5 to 10. | 10 to 15. | 15 to 20. | 20 to 30. | 30 to 40. | 40 to 50. | 50 to 60. | 60 to 70. | 70 to 80. | 80 to 90. | 90 to 100. | 100 to 110. | Total. |
|---|---|---|---|---|---|---|---|---|---|---|---|---|---|---|---|---|---|---|
| Still Born | 88 | 77 | 165 | | | | | | | | | | | | | | | 165 |
| Unknown | 28 | 13 | 10 | 2 | 3 | | 2 | 1 | 6 | 5 | 5 | 3 | 3 | 1 | | | | 41 |

TABLE NO. 3.

*Deaths for the Second Quarter of* 1856, *at fifteen distinct periods of life.*

| | |
|---|---|
| Under 1 year, | 532 |
| 1 to 2 | 228 |
| 2 to 5 | 327 |
| 5 to 10 | 134 |
| 10 to 15 | 51 |
| 15 to 20 | 69 |
| 20 to 30 | 278 |
| 30 to 40 | 232 |
| 40 to 50 | 160 |
| 50 to 60 | 119 |
| 60 to 70 | 116 |
| 70 to 80 | 102 |
| 80 to 90 | 44 |
| 90 to 100 | 10 |
| 100 to 110 | |
| | 2402 |
| Still Born | 165 |
| Total, | 2567 |

Included within the above table were 115 from the Blockley Alms House; 131 Blacks; 12 from the Pennsylvania Hospital; 3 from the County Prison; 1 from the Eastern Penitentiary, and 24 from the country, as follows:—

| | April. | May. | June. | Total. |
|---|---|---|---|---|
| Almshouse, | 45 | 44 | 26 | 115 |
| Blacks, | 47 | 45 | 39 | 132 |
| Pennsylvania Hospital, | 2 | 4 | 6 | 12 |
| County Prison, | | 2 | 1 | 3 |
| Eastern Penitentiary, | | | 1 | 1 |
| Country, | 9 | 9 | 6 | 24 |
| | 103 | 104 | 79 | 286 |

The following table furnishes the number of deaths for each week, during the quarter, together with the sexes, the adults and children.

| | | Male. | Female. | Adults. | Children. | Total. |
|---|---|---|---|---|---|---|
| 1856. | From March 29th to April 5th, | 104 | 109 | 96 | 117 | 213 |
| " | April 5th to April 12th, | 110 | 108 | 95 | 123 | 218 |
| " | April 12th to April 19th, | 116 | 97 | 90 | 123 | 213 |
| " | April 19th to April 26th, | 99 | 90 | 91 | 98 | 189 |
| " | April 26th to May 3d, | 118 | 101 | 94 | 125 | 219 |
| " | May 3d to May 10th, | 92 | 99 | 83 | 108 | 191 |
| " | May 10th to May 17th, | 90 | 104 | 88 | 106 | 194 |
| " | May 17th to May 24th, | 89 | 87 | 69 | 107 | 176 |
| " | May 24th to May 31st, | 87 | 88 | 77 | 98 | 175 |
| " | May 31st to June 7th, | 108 | 89 | 68 | 129 | 197 |
| " | June 7th to June 14th, | 85 | 74 | 56 | 103 | 159 |
| " | June 14th to June 21st, | 111 | 101 | 90 | 122 | 212 |
| " | June 21st to June 28th, | 111 | 104 | 67 | 148 | 215 |
| | Total, | 1320 | 1251 | 1064 | 1507 | 2571 |

